# Systematic review of ototoxic pre-surgical antiseptic preparations – what is the evidence?

**DOI:** 10.1186/s40463-018-0265-z

**Published:** 2018-03-01

**Authors:** Shubhi Singh, Brian Blakley

**Affiliations:** Division of Otolaryngology-Head and Neck Surgery, University of Manitoba, Health Sciences Centre GB421, 820 Sherbrook Street University of Manitoba, Winnipeg, MB R3T 2N2 Canada

**Keywords:** ototoxicity, hearing loss, antiseptic, vestibular dysfunction, chlorhexidine, iodine, povidone, ethanol, hydrogen peroxide

## Abstract

**Objective:**

There is uncertainty regarding the safety of surgical antiseptic preparations in the ear. A systematic review of the literature was conducted to assess the evidence regarding ototoxicity of surgical antiseptic preparations.

**Methods:**

A literature search was conducted using the PRISMA methods. Key words included “ototoxicity” “hearing loss”, “antiseptic”, “surgical preparation”, “tympanoplasty”, “vestibular dysfunction”, “chlorhexidine”, “iodine”, “povidone”, “ethanol”, and “hydrogen peroxide” using Medline, Embase, Cochrane Library, Scopus and Web of Science. We included peer-reviewed papers that 1) objectively measured ototoxicity in humans or animals through hearing, vestibular function or histologic examination, 2) studied topically applied surgical antiseptic preparations, 3) were either in English or had an English abstract. We excluded papers that were 1) in vitro studies, 2) ear trauma studies, 3) studies of ototoxic ear drops intended for therapy, or 4) case reports. Studies included in the final review were screened using the PRISMA method.

Current systematic review registration number pending: 83,675.

**Results:**

Fifty-six papers were identified as using PRISMA criteria. After applying our exclusion criteria, 13 papers met overall study criteria. Of these, six papers reported ototoxicity of iodine based solutions, five papers reported ototoxicity of chlorhexidine and ethanol and two papers assessed hydrogen peroxide. All papers reviewed were animal studies. Iodine based solutions show least harm overall, while chlorhexidine and high concentrations of alcohol based solutions showed most harm. The evidence on hydrogen based solutions was inconclusive.

**Conclusions:**

The overall evidence for anyone antiseptic solution is weak. There is some evidence that iodine, chlorhexidine, hydrogen peroxide and alcohol based antiseptics have ototoxicity. Conclusive evidence for human ototoxicity from any solution is not strong.

## Background

Antiseptic cleaning of skin prior to surgical intervention is the standard of care globally. Pre-surgical antiseptic preparation has been known to reduce the number of wound infections when used adequately [1]. However some standard antiseptic preparations have been shown to cause toxicity to the eyes and ears when used in head and neck surgery [2]. Currently, in otologic surgery, there remains uncertainty regarding the safety of surgical antiseptic preparations in the ear. This has been a long standing area of concern as described in a case series conducted by Bicknell et al. in the early 1960s. Bicknell et al. describe varying degrees of morbidity following tympanoplasty surgery, ranging from high frequency hearing loss to “dead ears” with the main commonality between patients being pre-surgical preparation of the ear with chlorhexidine [3]. The purpose of this study was to conduct a systematic review of the literature to assess the evidence regarding ototoxicity of standard surgical antiseptic preparations. The focus of this study was to review ototoxicity of povidone-iodine, chlorhexidine gluconate, ethanol and hydrogen peroxide.

## Methods

A systematic literature review was conducted using various combinations of the following key words: “ototoxicity”, “hearing loss”, “antiseptic”, “surgical preparation”, “tympanoplasty”, “vestibular dysfunction”, “chlorhexidine”, “iodine”, “povidone”, “ethanol”, and “hydrogen peroxide” using the databases: Medline, Embase, Cochrane Library, Scopus and Web of Science through September 2016. Further studies were obtained through screening references from relevant articles and the authors’ own databases and grey literature including legal proceedings. Criteria for inclusion of a published article in this review were applied to the collected studies by two independent reviewers.

Studies included were peer-reviewed papers that 1) objectively measured ototoxicity in humans or animals through hearing, vestibular function or histologic examination, 2) studied topically applied surgical antiseptic preparations, 3) were either in English or had an English abstract. Excluded studies were 1) in vitro studies, 2) ear trauma studies, 3) studies of ototoxic ear drops intended for therapy and 4) case reports. Studies included in the final review were screened using the PRISMA method [4].

Each paper identified through PRISMA criteria was reviewed for the following data items including: experimental subjects, solutions and concentrations tested and objective measure of ototoxicity. Objective measure of ototoxicity was defined as having any of the following: audiological or vestibular testing done before and after exposure to the solution, histological examinations or gross pathologic examinations. Due to the broad variation in objective measures of ototoxicity, no direct meta-analysis of the data was conducted between studies. However, the data obtained from the final results of studies meeting the set criteria in all studies are summarized in Tables 1, 2 and 3. Sources of error for these studies are further assessed in the discussion section.

**Table 1:** Results for iodine-based antiseptic preparations

Author, Year	Population	Intervention	Control	Outcome
J Aursnes, 1982 [5]	28 Guinea Pigs. Baseline Preyer’s reflex measured in all study animals.	Solutions tested: a) Iodine in 70% alcohol b) Iodophor in 70% alcohol c) Iodine in aqua dest. d) Iodophor in aqua dest. Exposure time 10, 30 or 60 minuets. Histopathology assessed after 2 weeks	Contralateral ear to experimental ear	a) Gross examination of the ear showed no mucosal changes after 10 mins of exposure for Iodine or Iodopher in aqua dest. b) Middle ear mucosal damage worst for solutions in 70% alcohol. c) Cochlear damage with exposure time of 60 min with Iodophor in 70% alcohol. d) Vestibular damage seen with Iodophor in 70% with exposure times of 30-60min
T Morizono, 1982 [7]	30 Chinchilla	Solutions tested: a) Povidone-iodine Solution at 1:10 dilution (1.0% available iodine) b) Povidone-iodine Scrub* at 1:10 dilution (0.75% available iodine) c) Povidone-iodine Scrub at 1:100 dilution Exposure time 10mins Effect on Compound Action potential (CAP) tested 2 hours, 24 hours post exposure *Scrub contains detergent	Contralateral ear to experimental ear	a) Povidone-iodine Scrub more toxic to cochlear function then solution b) Evoked action potential measure at round window, no change 2 hours after exposure with 1:10 dilution of Iodine Solution c) Evoked action potential measure at round window 2 hours after exposure with 1:10 dilution of Iodine Scrub caused severe depression at all tested frequencies (2, 4, 8, 12kHz) d) Increased toxicity with increased concentrations for both solution and scrub
T Morizono, 1983 [8]	30 Chinchilla	Solutions at different dilutions d) Povidone-iodine Solution at 1:10 dilution (1.0% available iodine) e) Povidone-iodine Scrub* at 1:10 dilution (0.75% available iodine) f) Povidone-iodine Scrub at 1:100 dilution Duration of exposure time 10mins Effect on Compound Action potential (CAP) tested 2 hours, 24 hours post exposure *Scrub contains detergent	Contralateral ear to experimental ear	a) High frequency losses (8 and 12kHz) after 10, 30 and 120min exposure to iodine scrub at 1:100 dilution
T. Inchibangase, 2011 [6]	70 Guinea Pigs – Divided into groups based on age (infant, young and adult)	Solution tested a) Povidone-iodine 10% solution b) Povidone-iodine scrub Effect on Compound Action potential (CAP) tested at 24h, 7days and 28days	Contralateral ear to experimental ear	a) No action potential at 24hours after application of Povidoneiodine scrub b) 24 hours after exposure to povidone-iodine solution, significant ototoxicity measured in infant group, less in young and least in adult c) 8 fold dilution of povidoneiodine solution showed no hearing loss in adults, loss for young at 2 and 4kHz d) Showed aged related variation associated with ototoxicity in guinea pigs
M. Ozkiris [16]	24 Sprague-Dawley Rats	Solution tested a) 5, 7.5, 10% Povidone-iodine solutions No exposure time given. Otoacoustic emissions measured at 1 and 10 days after exposure	Contralateral ear to experimental ear	a) At 5% concentration some statistically significant decreased hearing at day 1 but resolved by day 10 b) At 7.5% and 10%, day 10 results showed decreased in hearing in frequencies ranging from 1.5-12kHz
R. Yagiz, 2003 [17]	7 adult guinea pigs	Solution Tested a) Povidone-iodine 10% solution No exposure time given. Hearing tested at 10 days and 4 weeks after exposure	a) Saline as a negative control in the contralateral ear to experimental ear in 4 animals b) Gentamycin as a positive control the contralateral ear of 3 experimental animals	a) 4 animals could not be tested due to severe oedema of the external auditory canal b) No Otoacustic emissions present 10days or 4 weeks after exposure

**Table 2:** Results for chlorhexidine and ethanol-based antiseptic preparations

Author, Year	Population	Intervention	Control	Outcomes
R. Perez, 2000 [10]	25 Sand Rats	a) Solutions tested Povidone-iodine 10% solution b) Chlorhexidine Gluconate 0.5% solution c) Ethyl alcohol in 70% aqueous solution Exposure time 3 days ABR and vestibular evoked potentials (VsEP) testing 8 days after initial exposure	a) Saline as negative control b) Gentamycin as positive control	a) ABR not present after Chlorhexidine Gluconate 0.5% solution b) ABR present at baseline after application of Povidone-iodine 10% solution, VsEP present in all test animals c) No ABR or VsEP recorded in 2/5 animals after Ethyl alcohol 70% solution, 3/5 had elevated thresholds (70-80 dB) d) Erythema and oedema noted in 5/5 middle ear cavities after application of ethyl alcohol
Y. Igarashi, 1985 [9]	12 Cats	Solutions tested a) Chlorhexidine Gluconate 2.0% b) Chlorhexidine Gluconate 0.05% Exposure time every 2 days × 3 applications. Histologic examination at 7 days and 4 weeks	Contralateral ear to the experimental ear	a) Gross examination of middle ear space showed thick serous fluid retention in12/12 animals b) Histological examination showed loss of outer hair less in lower cochlear turns, with 85% loss near the round window after application of Chlorhexidine gluconate 2% solutions c) Little to no damage to the outer hair cells seen with Chlorhexidine 0.05% solution
J. Aursnes, 1981 [14]	48 Guinea Pigs	Solutions tested a) Chlorhexidine 0.1% in 70% alcohol b) Chlorhexidine 0.1% in aqua. Solution c) Chlorhexidine 0.5% in 70% alcohol d) Chlorhexidine 0.5% in aqua. Solution Exposure time 10, 30 and 60 mins. Histological examination done after 2, 3, 4 or 10 weeks post exposure	Contralateral ear to experimental ear	a) Gross examination showed extensive fibrotic tissue after 60 min exposure time b) Greater degree of fibrosis with 0.5% solution compared to 0.1% solution c) Total destruction of outer hair cells seen 3 weeks after exposure with Chlorhexidine 0.5% in 70% alcohol
H. G. Galle, 1986 [18]	2 Guinea Pigs	Solution tested a) Savlon™* in 1:100 dilution Cochlear microphonics, histologic examination and behaviour measured at 24 h and 48 h after exposure * Composed of 1.5% Chlorhexidine Gluconate and 0.15% cetrimide (quaternary ammonium compound)	Contralateral ear to experimental ear	a) Severe vestibular dysfunction based on behaviour but effects diminished after 24 h and again after 48 h b) Hearing thresholds increased from baseline of 35 dB to 70 dB SPL
T. Morizono, 1981 [11]	23 Chinchillas	Solutions tested a) Ethanol in contractions of 0.1, 1, 3, 10, 25, 50, 70 and 100% Exposure time was 10mins, 24 h. Cochlear mircophonics and endocochlear Action potentials tested	Contralateral ear to experimental ear	a) Variable outcomes with some animals showing decrease in all frequencies tested with cochlear microphonics with 3% solutions while others showed no deficits with 50% solution

**Table 3:** Results for hydrogen peroxide-based antiseptic preparations

Author, Year	Population	Intervention	Control	Outcome
R. Perez, 2003 [13]	22 Sand Rats	a) Hydrogen peroxide 3% solution Exposure time 5 days. ABR and VsEP tested on day 8 after initial exposure	a) Saline as negative control b) Gentamicin as positive control	a) ABR could not be tested in 3/12 animals. All remaining animals had elevated base line from 55 dB to 108 dB b) VsEP could not be recorded in 5/12 animals. All remaining animals had a increased from baseline of mean latency time
ME Nader, 2007 [12]	18 Chinchillas	Solution tested a) Hydrogen peroxide 3% solution Exposure time 5 min ABR performed 1 day after exposure	Contralateral ear to experimental ear	b) No difference of ABR from baseline recording prior to instillation of hydrogen peroxide.

## Results

**Fig. 1:**
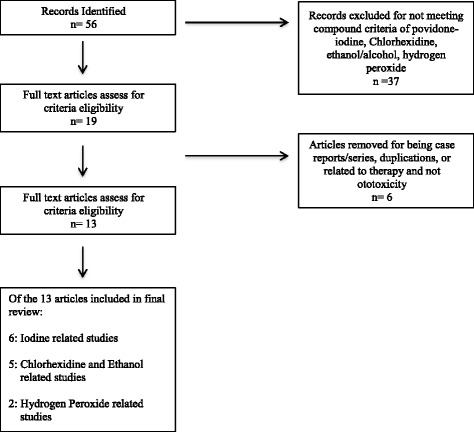
Flow chart of reviewed articles

Fifty-six studies were identified through database searches and searches of relevant article references. Using pre-set criteria as mentioned above, 43 articles were eliminated as outlined in Fig. 1. Of the final 13 articles included in this review; six pertained to iodine based solutions, five to chlorhexidine and ethanol and two papers to hydrogen peroxide. All papers identified were animal studies.

Of the papers assessing the ototoxicity of povidone-iodine, Aursnes et al. found that povidone-iodine solutions in 70% alcohol with greater then 10 min of middle ear exposure to the solution caused an increase in cochlear damage [5]. Ichibangase et al. assessed ototoxicity of povidone-iodine 10% solution in guinea pigs of varying ages [6]. They found that those animals deemed to be infant or young had increased cochlear toxicity compared to adult guinea pigs. One reason they suggested for this finding was increased permeability of the round window membrane in infant versus adult guinea pigs as the membrane thickens with age [6]. Of the studies pertaining to povidone-iodine scrubs that contain detergents, all studies found that scrubs caused higher ototoxicity than povidone-iodine solutions, suggesting that detergent facilitates entry of the scrub into the inner ear [6–8].

In studies assessing chlorhexidine gluconate solutions, Igarashi et al. found that a concentration of 0.05% caused no change in Auditory Brainstem Response (ABR) from baseline after three applications of solution to the middle ear [9]. Perez showed that after three applications of 0.5% chlorhexidine gluconate to the middle ear of sand rats, no ABR were present in previously normal hearing animals [10]. Finally, three applications of chlorhexidine solution at 2.0% concentration caused destruction of outer hair cells on histological examination of the cochlea. Concentrations of 0.05 and 2.0% were shown to cause thick serous middle ear discharge on gross pathological examination. Similarly,. Perez et al. found that 70% Ethyl Alcohol caused gross pathological changes to the middle ear space including erythema and edema. In some animals oedema of the external ear canal was so severe that testing of hearing was not possible [10]. Morizono et al. tested several strengths of ethanol ranging from 0.1 to 100% pure ethanol in the middle ear cavities of chinchillas [11]. They concluded that there was evidence of ototoxicity for ethanol concentrations greater than 10% using cochlear microphonics [11].

Finally, Perez et al. and Nader et al. assessed the ototoxicity of 3% hydrogen peroxide solutions [12]. While Nader et al. found no difference in ABR from baseline after a 5 min exposure of 3% hydrogen peroxide to the middle ear of chinchillas, Perez et al. found the majority of sand rats tested had an increase in threshold from an average of 55 dB to 108 dB after 5 applications of 3% hydrogen peroxide [12, 13].

## Discussion

In this review, we identified 13 studies showing the ototoxicity of povidone-iodine, chlorhexidine gluconate, ethanol/ ethyl alcohol and hydrogen peroxide in controlled non-trauma settings. All studies were animal studies and no direct human correlation can be drawn given the differences in anatomy of the middle ear space, dosing of antiseptic preparations and in some cases the duration of exposure being in the order of several weeks. However, some solutions showed high ototoxicity in relatively low concentrations and short exposure times. This includes povidone-iodine scrub which contains detergent, povidone-iodine in 70% alcohol, and chlorhexidine gluconate in 70% alcohol [5–7, 14]. However for other solutions there is no consensus from the studies identified. (Tables 1, 2 and 3).

There are several limitations of this current review. The methods and objective measures are inconsistent.

All the studies identified in this review were animal studies so we are cautious about drawing conclusions from different species using different methods on the potential of the solutions to cause damage in human subjects. In studies conducted on guinea pigs and chinchillas the main hypothesized method of inner ear penetration for solutions is through the round window. The Chinchilla round window membrane is 1/6 of the thickness of that of humans therefore this model is likely over estimating ototoxicity in humans [15].

There are also several challenges differentiating conductive hearing loss from sensorineural hearing loss in animal subjects. The time period over which animals were assessed may not have been adequate [7].

## Conclusion

Given the findings of this review, the evidence of human ototoxicity of currently used antiseptic preparations is not strong. Iodine based, non-alcoholic, non-detergent solutions may be the least ototoxic but all should be used with caution.

## Abbreviations

ABR: Auditory Brainstem Response
CAP: Compound Action Potential
PRISMA: Preferred Reporting Items for Systematic Reviews and Meta-Analysis
SPL: Sound Pressure Level
VsEP: Vestibular Evoked Potential
